# Phenolic Compounds in Honey and Their Relationship with Antioxidant Activity, Botanical Origin, and Color

**DOI:** 10.3390/antiox10111700

**Published:** 2021-10-27

**Authors:** Ana L. Becerril-Sánchez, Baciliza Quintero-Salazar, Octavio Dublán-García, Héctor B. Escalona-Buendía

**Affiliations:** 1 Food and Environmental Toxicology Laboratory, Faculty of Chemistry, Universidad Autónoma del Estado de México, Toluca 50120, Mexico; albecerrils@uaemex.mx; 2 Faculty of Tourism and Gastronomy, Universidad Autónoma del Estado de México, Toluca 50200, Mexico; 3 Sensory Evaluation and Consumer Studies Laboratory, Biotechnology Department, Universidad Autónoma Metropolitana, Mexico City 09340, Mexico; hbeb@xanum.uam.mx

**Keywords:** honey, polyphenols, antioxidants, flavonoids, honeybee

## Abstract

Honey has been employed since antiquity due to its sensory, nutritional, and therapeutic properties. These characteristics are related to its physical and chemical composition. For example, phenolic compounds are substances that can determine antioxidant activity, as well as sensory characteristics, and can be employed as biomarkers of floral and geographical origin. This has generated a growing interest in the study of phenolic compounds and their influence in the intrinsic properties of this beekeeping product. This review aims to summarize, analyze, and update the status of the research that demonstrates the role of phenolic compounds in antioxidant activity, botanical-geographical origin, and the sensory characteristics of honey. These phenolic compounds, according to various results reported, have great relevance in honey’s biological and functional activity. This leads to research that will link phenolic compounds to their floral, geographical, productive, and territorial origin, as well as some sensory and functional characteristics.

## 1. Introduction

Honey is a natural sweet and homogenous substance produced by honey bees from the nectar or secretions of plants or excretions of plant sucking insects on the living parts of plants, which the bees collect and transform [1]. It is composed of 80–85% mix of sugars, 15–17% water, 0.1–0.4% proteins, and, to a lesser degree, other compounds such as enzymes, organic acids, vitamins, and phenolic compounds, all of which determine its sensory and functional characteristics [2]. Its consistency may be fluid, viscous or crystalline, depending on the quantity and type of sugars present, moisture content, and storage temperature [3]. The color can be white, yellow, brown, among others, and is determined, in part, by the presence of phenolic compounds, flavonoids, and minerals [4]. The development of the color is also linked to monosaccharide content, which are the sugars that are found in the highest percentage and are responsible for some other sensory and functional properties such as flavor, texture, moisture retention, shelf life, conservation, among others [2]. Also, the physical and biochemical properties of honey have generated great interest, due to its health-promoting capacity, as well as its anti-inflammatory, antioxidant, antimicrobial, anticarcinogenic, anti-diabetic properties, among others [5,6].

These physical, biochemical, and sensory properties are influenced by its botanical origin, the geographical area, the environment, as well as floral and entomological sources, and processing factors. Honey can be classified, concerning its botanical origin, as *monofloral* (when it is produced from the nectar o honeydew of a single botanical species or if its presence is the prevalent one) and multifloral (when it comes from more than one botanical species) [7]. Honeydew is the honey, which comes mainly from excretions of plant sucking insects on the living parts of plants or secretions of living parts of plants [1].

Moreover, the global production of honey exhibits a positive tendency [8]. In 2019, world production of honey was 1,850,868 tons. China was the highest producer, with 457.2 thousand tons, followed by Turkey, with a production of 114.11 thousand tons, and Argentina, with 79.47 thousand tons [9]. Its production and commercialization have focused on fulfilling the expectations of the market, providing clear information concerning its physicochemical, sensory, and nutritional qualities. For this purpose, various studies have been carried out, concerning said characteristics of the varieties of local and imported honeys [10].

The honey industry has had to comprehend the behavioral tendencies of the consumers. The aspects of quality contemplated during the selection of one of the many types of honey on the market from appearance, sensory properties, safety, botanical origin, nutritional value, brand reputation, as well as environmental and ethical factors [11,12]. This search for quality reflects, with a greater tendency, the awareness that has developed among consumers, concerning the importance of diet, with regard to health and well-being [12].

The beneficial effects of honey on human health is mainly due to its content of phenolic compounds [6]. Said compounds, besides being bioactive components, are biomarkers of origin and determine some sensory characteristics, which has led to a considerable amount of research focused on the study of phenolic compounds present in this beekeeping product. Thus, this review aims to summarize, analyze, and update the status of the research in the last decade, which demonstrates the role of phenolic compounds in antioxidant activity, its botanical and geographical origin, and the color of honey.

## 2. The Phenolic Compounds of Honey

Phenolic compounds are substances that contain phenol (hydroxybenzene) within their structure, bonded to aromatic structures (unsaturated cyclical structures) or aliphatic structures (branched structures that do not form rings) [13]. They are considered the main group of secondary metabolites from plants, and their presence in the animal kingdom is due to their ingestion of these [14].

The phenols present in foods are mainly classified as simple phenols, coumarins, quinones, betacyanin, lignans and lignin, acidic phenols, flavonoids and tannins; these last three are the main dietary phenolic compounds [13].

Phenolic acids are characterized by the presence of at least one hydroxyl group and are functional derivatives of benzoic acid (C_6_▬C_1_) or cinnamic acid (C_6_▬C_3_). The basic chemical compounds derived from benzoic acid are p-hydroxybenzoic, protocatechuic, gallic, vanillic, syringic, salicylic and gentisic acids. Moreover, hydroxycinnamic acids are basic precursors in the biosynthesis of various vegetable phenols. The basic chemical compounds derived from cinnamic acid are p-coumaric, caffeic, ferulic, and synaptic acid [15].

Flavonoids consist of three rings. The first a dehydroxylated phenolic ring, the second is a monohydroxylated phenolic ring, which can be ortho-bihydroxylated or vic-trihydroxylated, and the third heterocyclic ring with pyran oxygen. These are classified according to the oxidation state of the third ring and the position of the secondary ring. If the phenolic ring is found in position “2”, it gives way to flavones, flavanols, dihydroflavanols, catechins, flavans, and anthocyanidins; in position “3”, to isoflavonoids; in position “4”, to 4-phenyl-coumarins and neoflavonoids. Flavonoids are considered the most widely distributed polyphenols in plants, with more than 5000 compounds. The most common are quercetin, kaempferol, and myricetin [13,15,16].

Honey contains phenolic compounds because, during its production, the bees mix their body fluids with the nectars of flowers or secretions of plants which are composed of water, sugars, proteins, and phenolic compounds [17]. These compounds have shown significant biological activity in the treatment of various diseases, which has generated a large number of studies [6,18,19,20,21,22]. Honey has been shown to have anti-cancer effects, and some studies have focused on entering its mechanism [23,24,25]. For example, it has been shown that Tualang honey, in the treatment of breast cancer, modulates hematologic, estrogenic and apoptotic activities, in experimental animal studies [26]. In addition, it is suggested that it could be an adjuvant for the chemotherapeutic agent, due to the protection it can provide to non-cancerous cells from the toxic effects of tamoxifen by increasing the efficiency of DNA repair mechanism in cells [27].

Another property of interest in honey is that of being anti-diabetic, because it has been observed that it has the ability to inhibit the enzyme alpha-amylase [28,29]. Likewise, its potential in the treatment of glycemia has been studied, since it has been observed that it reduces blood glucose after consumption [21,30,31]. In addition, it has been used as a therapeutic agent in the treatment of diabetic wounds [32].

The study of honey has also been of great interest for its biological and clinical actions against chronic diseases mediated by inflammation, such as cardiovascular, neurodegenerative, and arthritic. The study of the bioavailability of phenolic compounds in honey has made it possible to have an application in the treatment of inflammatory pathways in disorders of the gastrointestinal tract, edema, cancer, metabolic and cardiovascular diseases, and gut microbiota [33]. The absorption of polyphenols in the upper gastrointestinal tract is relatively low, which is why they tend to accumulate in the colon, and they can change the intestinal ecology [34], some bacterial groups may be limited, others can thrive in the niche of the accessible biome impacting the health of the host [35].In this sense, it has been shown that phenolic compounds can improve the survival and adhesion capacity of probiotics in conditions similar to the gastrointestinal tract, improving a variety of biochemical markers and risk factors for chronic diseases [36]. The favorable effect of honey on the growth of intestinal beneficial bacteria (lactic acid bacteria), as well as its antibacterial action on pathogens, has been studied, which would suggest that the consumption of honey presents a probiotic and prebiotic activity that provides a benefit for gut health [33,37,38,39]. In Chinese buckwheat honeys, the phenolics and oligosaccharides seem to synergistically impact human intestinal microbes [40].

The interaction of phenolic compounds with the gut microbiota as well as beneficial microorganisms (probiotics) is an emerging factor in achieving the health-promoting effects induced by these compounds. Foods, such as honey, which contain phenolic compounds, prebiotics and have good interaction with probiotics, present an added value of great interest in various industries, such as the food industry due to their acceptability and health benefits [36]. Recent and intensive interest in the biological and clinical action of phenolic and flavonoid compounds in honey has generated endless studies that deserve to be analyzed in-depth in their own review.

On the other hand, it has been observed that the values of Total Phenol Content (TPC) and Total Flavonoid Content (TFC) also vary according to the country or region where the honey is produced, and not only by botanical origin (Table 1).

The determination of TPC is carried out via the Folin-Ciocalteu colorimetric assay, which measures the absorbance with a spectrophotometer at 750 nm [52]. The results are expressed as mg of gallic acid equivalents of honey (GAE/g) in most of the studies. However, some studies report results as mg GAE/g or mg GAE/kg; this discrepancy could be due to the national regulatory standards of the countries in which the determinations are carried out. For the determination of TFC, absorbance is measured at 510 nm. The values are expressed as quercetin equivalents per gram (QE/g) or milligram of catechin equivalents per kilogram [67].

The values of TPC and TFC according to the botanical origin have aroused an interest in studying the biological activities of monofloral honeys. It has been reported that extracts of jujube leaves (*Ziziphus jujuba*) have a great potential for inhibition of the enzymes α-amylase and α-glucosidase, among other biological capacities [68], which has generated an interest in the research of the biological activity of jujuba honeys [64,69,70].

The phenolic compound and flavonoid profiles of honey allow the evaluation of its quality, since it identifies emerging risks, facilitates the differentiation of the varietals, botanical origin, and detects adulteration and bioactive compounds with health-promoting properties [71]. The analysis of the profiles of phenolic compounds and flavonoids in honey is mainly carried out by High Performance Liquid Chromatography (HPLC) and Fourier-Transform Infrared Spectroscopy (FTIR). The predominant phenolic compounds found in several samples have been p-coumaric, caffeic, chlorogenic, protocatechuic, and vanillic acids, rutin, myricetin, apigenin and quercetin (Tables S1 and S2). In buckwheat (*Fagopyrum esculentum*) honey, a greater presence of flavonoids than of phenolic acids was reported to have been found [55]. In *Mimosa scabrella* Bentham honeydew, significant differences in the content of phenolic acids and flavonoids from various geographical regions were observed, probably due to the presence of flower nectars from each region [72]. Direct comparisons between honeys and their phenolic profiles are difficult since the extract, detection methodology and instruments, and the number of components determined are highly variable among published studies, but -in some cases- the presence of certain compounds according to the botanical origin can be observed.

The phenolic profile of honey has allowed the creation of a comprehensive vision of the health-promoting properties, attributed to its floral and geographical properties [73]. In various studies, myricetin, which is a flavonoid widely recognized for its nutraceutical value, for its anticancer, anti-diabetic, anti-inflammatory and antioxidant activity, has been detected, and it has been suggested that it protects against diseases such as Parkinson’s and Alzheimer’s [74]. Pinocembrin has antioxidant, antimicrobial, and anti-inflammatory properties. Its potential to inhibit histidine decarboxylase as a new candidate for natural antiallergic drugs has recently been studied [75].

In monofloral honeys of *Amorpha fruticosa* L., nine phenolic acids and their derivatives have been found, as well as twelve flavonoids, and, for the first time, the compounds chrysoeriol and formononetin [76]. The latter has been shown to be an active component with great potential for the prevention and treatment of chronic diseases, such as cancer, obesity, and neurodegenerative diseases [77].

In safflower honey (*Carthamus tinctorius* L.), quercetin and vanillic acid have been found to be the prevalent phenolic compounds. The anti-inflammatory and analgesic action of honey was attributed to vanillic acid through the antioxidation and the inhibition of the production of pro-inflammatory cytokines in extracts of Hemp honeys [78].

The health benefits of honey will depend on the bioavailability of the phytochemical compounds, their absorption, and metabolization methods [6]. For example, high molecular weight polyphenols are not absorbed in the small intestine due to their chemical complexity. Poor absorption causes these compounds to be detected in the systemic circulation at trace levels. However, it has been assumed that the beneficial effects of polyphenols may be related to the modulation of gut microbiota composition and activity or through metabolization of polyphenols into metabolites with greater bioavailability by the microbiota through multienzymatic reactions such as deglycosylation, dihydroxylation, decarboxylation, hydrogenation, sulfation, glucuronidation and C ring cleavage of the benzo-γ-pyrone system [34]. In general, the metabolic transformations of polyphenols are included in phase I (oxidation, reduction, hydrolysis, etc.) and phase II (conjugation) metabolism [35].

## 3. Antioxidant Activity of Honey

Antioxidants are compounds that protect cells from damage caused by free radicals; these capture, stabilize or deactivate reactive oxygen species (ROS) before they reach cells [79]. The cell’s antioxidant process, used to overcome the harmful effects of ROS, will depend on the origin of the cell. In animal cells, the mitochondria is the main source for ROS production, while in plant cells, it is the chloroplast, which generates singlet oxygen (^1^O_2_), superoxide (O_2_^•−^), and hydrogen peroxide (H_2_O_2_), which, together with the hydroxyl radical (HO^•^) and hypochlorous acid (HOCl), are the main reactive oxygen species [80,81]. In normal conditions, these free radicals have important functions, such as phosphorylation and cell signaling. When chain reactions of free radicals are amplified, which damages biomolecules, and the equilibrium between prooxidant and antioxidant substances is lost in favor of the former, this phenomenon is known as oxidative stress [82].

Oxidative stress produces cell damage, which leads to the manifestation of degenerative cardiovascular illnesses, as well as cancer and aging. To reduce these negative effects, the endogenous defense system produces antioxidant enzymes and non-enzymatic compounds, such as glutathione, vitamins, coenzyme Q, among others; however, it requires external help to modulate these negative effects with greater efficacy. Dietary antioxidant compounds are considered external aids (since they are obtained during the ingestions of foods); most of them are present in fruits, vegetables, and other sources from the plant and animal kingdoms [80].

Exogenous antioxidants are mainly found in fruits and vegetables. Some foods of animal origin contain them, due to a diet based on plant sources. The most commonly consumed ones are vitamins C and E, some trace elements (selenium, zinc, manganese, and copper), β-carotene and phenolic compounds, among others [83].

The antioxidant activity of phenolic compounds is attributed to their capacity to eliminate free radicals by donating hydrogen atoms, electrons or metallic cations; this capacity of interaction with free radicals is due to their structure (particularly due to the number and positions of the hydroxyl groups and the nature of the substitutions in the aromatic rings) [84]. Also based on the binding of these compounds to organic acids and sugars [85]. The antioxidant capacity can diminish in vivo according to the external source and the interaction with other sources, such as the number of nutrients that are digested, absorbed, and metabolized [83]. Because of the aforementioned, the total content and bioavailability of antioxidants in foods are important to determine their antioxidant capacity.

The antioxidant activity of phenolic compounds can be an alternative to promote the maintenance and recovery of the balance of the intestinal microbiota since these compounds can stimulate the secretion of antioxidant enzymes such as superoxide dismutase (SOD), catalase (CAT), glutathione peroxidase (GPx), glutathione reductase (GR) and peroxiredoxins that blocking on ROS or stimulating endogenous defense system [86].

Phenolic compounds are the ones mainly responsible for the antioxidant activity of honey. The oxidative reactions are very complex, which causes the prediction of the antioxidant capacity of honey to perhaps seem inadequate when applying a sole assay, which is why Pentós et al. [87] recommend using at least two methods to increase the reliability of the experiment.

On the other hand, the most frequently employed methods for determining the antioxidant activity in honey are 2,2′-azino-bis-3-ethylbenzothiazolin-6-sulphonic acid (ABTS), 2,2-diphenyl-1-picrylhydrazyl free radical (DPPH), and Ferric Ion Reducing Antioxidant Power (FRAP). The ABTS method expresses the antioxidant activity of the sample in contrast to the concentration of antioxidants, which may include the proportion of biologically inactive antioxidants. The results are expressed as mg of ascorbic acid equivalents per 100 g of honey (AAE/100 g). DPPH is another assay method frequently employed for the analysis of antioxidant activity in honey, since it is very simple and fast; it shows the global antioxidant capacity of the sample, employing both solid and liquid samples [88]. The total of antioxidants is determined, based on the scavenging activity against the DPPH free radical, through the IC50 parameter, which represents the concentration of material needed to inhibit 50% of the free radicals [89]. Another assay employed is the FRAP determination, which estimates the capacity of the compound to reduce the Fe^3+^/Fe^2+^ pair. It uses antioxidants as reducers in a colorimetric method. The results can be expressed as the concentration of antioxidants with ferric reducing power equivalent to 1 μM. High values of FRAP indicate a high reduction of the Fe^3+^/Fe^2+^ ions, which correspond to strong antioxidant properties [90].

In various studies, a high correlation between TPC and TFC with the antioxidant capacity of honeys has been reported [47,52,91,92]. For example, Argentine honeys [66] were reported to have a higher correlation with phenolic content (*r =* 0.91) than with flavonoid content (*r =* 0.51). The opposite case was observed in Malaysian honey, whose correlation with antioxidant activity (FRAP) was higher with TFC (*r =* 0.408), than with TPC (*r =* 0.385) [42]. However, some studies report a low or negative correlation, which indicates that, in some honeys, their antioxidant capacity does not depend solely on phenolic compounds and could be due to organic acids, amino acids, proteins, as well as to Maillard reaction products [42]. Al-Farsi et al. [89] relate negative correlation to the calculation of (IC50) in the DPPH assay, where negative values indicate higher antioxidants in Omani Honey (−0.616). A similar case was reported for European honeys (TPC *r =* −0.319; TFC *r =* −0.386) [45].

In extracts or fractions of honey, the method and solvents employed produce different concentrations of phenolic compounds and antioxidant activity. Studies reported the extracts obtained with the solvent ethyl acetate presented a higher recovery of phenolic compounds and also observed an increase in antioxidant and biological activity than with diethyl ether [25], *n*-hexane, chloroform, ethyl acetate and, *n*-butanol [47]. A similar case presented itself, according to conservation treatments. Manuka honey, processed by high pressures (HHP), presented a significant increase (*p* < 0.05) in antioxidant capacity when compared to unprocessed honey (52.36 ± 0.03%) [93]. With regard to thermic treatments for jujube honeys, a linear increase was reported in antioxidant activity in samples with heating to 45 °C and 55 °C and a logarithmic increase to 65 °C, besides a strong correlation between the total phenol content and antioxidant activity (45 °C *r* = 0.980, 55 °C *r* = 0.988, 65 °C *r* = 0.996) [94].

## 4. Phenolic Compounds as Indicators of the Botanical Origin of Honey

In honey production, climate, environmental conditions, and botanical origin are considered part of the quality parameters, since they determine not only its physicochemical composition, but also its sensory, nutritional, bioactive, and commercial value. Monofloral honeys usually have a higher retail price than the multifloral varieties [95].

Botanical origin is traditionally evaluated by melissopalynology analysis which microscopically identifies pollen origin using reference slides and a pollen atlas. It provides reliable information on the floral types that are the source of obtaining the pollen used by bees to produce honey. However, several authors report that it has proven to be complicated and laborious since it requires a lot of time and highly qualified personnel, in addition to the fact that it cannot detect the fraudulent addition of pollen [96,97]. Due to the above, in recent years alternative methodologies have emerged for the determination and authentication of the botanical origin of honey.

Spectrophotometric methods are widely used for the determination of polyphenols and flavonoids in honey because they are fast and non-invasive [98]. To mention a few, Fourier-transform infrared spectroscopy (FTIR) allows to discriminate and authenticate honeys since it has the potential to provide information on their compounds and functional groups. However, it is necessary to process the data using chemometric tools such as main component analysis, partial least squares, and discrimination analysis [99]. Through the methodologies Headspace solid-phase microextraction coupled with gas chromatography-mass spectrometry (HS-SPME/GC-MS) allows the floral origin to be identified by employing the construction of a barcode-type identifier that differentiates the honey by identifying the individual components of the fraction obtained, as presented in Polish honey [100]. Elemental profiles by Energy Dispersive-X Ray Fluorescence are a suitable screening technique that allows the pre-classification of monofloral and multifloral honeys concerning their botanical origin. The samples do not need any treatment for the analysis, which increases the performance, however, a pre-concentration is needed to reduce their limits of quantification, which can increase the complexity of the analysis [101]. There are other spectrophotometric methods used for the analysis of the authenticity of honey such as nuclear magnetic resonance (NMR), thin layer chromatography (TLC), liquid chromatography (LC), anion exchange chromatography (HPAEC), stable isotope ratio mass spectrometry (IRMS), and electroanalytical methods that are discussed in detail in the review by Trifković et al. [98].

The analyzes of artificial senses, together with chemometric, are fast and reliable tools for discrimination since they allow honeys to be classified according to their botanical origin. The downside is that you must know how to merge the data from each device. In this sense, in unknown samples of Sicilian honeys, the botanical origin could be identified with e-tongue, computer vision system, and multivariate statistical analysis [96].

The use of omics technologies such as genomic tools next-generation sequencing (NGS), DNA barcoding, among others have been proposed for the determination of plants, pollen, botanical, and geographical origin of honey, as it is faster and more reliable than melissopalynology analysis, because honey contains traces of the bee and plant of origin DNA, which are highly efficient and precise markers for the identification of species [102]. In honeys from northeast India, the DNA metabarcoding study identified 74 numbers of polliniferous plant species compared to the melissopalynological study that identified 76 numbers, concluding that the two techniques are important and relevant for identifying polliniferous plants. This discrepancy occurred because some pollens were not identifiable or because of an incomplete plant database, which suggests strengthening the information on the plant DNA barcode genes [97].

Phenolic compounds have a strong relation to the floral origin of the honey. In this sense, the analysis of the phenolic compounds constitutes a technique for the geographical and floral evaluation.

The abundance of flowering determines the phenol content in honey, it changes throughout the year. Some authors reported that in certain seasons of the year, flowering is so scarce that beekeepers have to feed the bees with sugar syrup, which leads to a decrease in the content of phenolic compounds and other elements [103]. The temporality of the flowering produces fluctuations in the nectar available for the production; the more homogenous the nectar, the higher the stability of the chemical composition of the honey [52].

In various studies, a higher variation in phenolic compound content in multifloral has been observed. Kavanagh et al. [95] reported that, in multifloral Irish honeys, significant variations in TPC were found, ranging from 2.59 to 81.10 mg GAE/100 g of honey (n = 124, Mean ± SD = 23.84 ± 13.07). They mention that these variations could be due to the harvest time and the context of the landscape. In honeys produced in rural areas, a significantly lower value of total phenolic content was registered (20.32 mg GAE/100 g), in contrast to urban areas (28.26 mg GAE/100 g), due to the fact that, in Irish urban areas, there is a greater diversity of floral resources.

The diversity in flowering for the production of multifloral honey could increase the values of phenolic compounds. In Kosovo, Ibrahimi & Hajdari, [104] observed a lower TPC in the multifloral acacia (25.76 ± 10.16 mg GAE/100 g) and chestnut (35.77 ± 8.26 mg GAE/100 g) honeys, but with a lower variation among samples, compared to the multifloral from meadows (46.48 ± 16.59 mg GAE/100 g) and forests (46.33 ± 18.95 mg GAE/100 g). Muñoz et al. [56] reported that the wild multifloral of Peru registered a higher phenolic compound content with a lower variation among samples (207.89 ± 2.18 mg GAE/100 g), compared to monofloral eucalyptus honeys (83.15 ± 4.09 mg GAE/100 g).

However, the content of phenolic compounds is closely related to the botanical origin of honey, which is also a determining factor in its biological functions [95]. There are also monofloral honeys which present a high phenolic compound content, thus generating great interest in these biocomponents. Various studies show that the monofloral honey with the highest phenolic compound content, reported until now, is Manuka honey (*Leptospermum scoparium*), from New Zealand or Australia (217.0–203 mg GAE/100 g) [54]. This high content also confers a high antioxidant and antibacterial capacity, which allows it to be considered a medicinal honey. This has led to the use of Manuka honey as a reference for the comparison of bioactive compounds with others of a distinct botanical and geographical origin [54,87,92,95,105,106,107]. Regarding the comparison of the content of phenolic compounds of Manuka honey with others, such as Polish honeys, no significant differences were found (*p* ≤ 0.05) in the TPC of buckwheat honey (211.0 ± 11.4 mg GAE/100 g) and honeydew (201.0 ± 9.9 mg GAE/100 g), compared to the values of the commercial Manuka honey. In contrast, the Polish multifloral (141.0 ± 7.3 mg GAE/100 g) and linden honey (133.1 ± 5.4 mg GAE/100 g) did present significant differences [54]. Some of the most relevant factors that contribute to the antimicrobial power of honey are phenolic acids and flavonoids [108]. Some Polish honeys from Buckwheat (*Fagopyrum esculentum* Moench), Thyme (*Thymus vulgaris* L.), Cornflower (*Centaurea cyanus* L.), and Tansy phacelia (*Phacelia tanacetifolia* Benth) showed greater activity against *Pseudomonas aeruginosa* and inhibition in the growth of *Staphylococcus epidermidis* compared to Manuka honey [109]. It has been reported that Tualang honey from Malaysia presents higher values of phenols and flavonoids than Manuka honey, and it is also more effective against some gram-negative bacteria and in the treatment of burn wounds [105]. A similar case was observed in other Malaysian honeys, where a significant difference (*p* < 0.05) was observed between the TPC and TFC of Logan and Sourwood honeys that presented higher values than Manuka honey, while only Sourwood honey (653.75 μM Fe (II)/100 g) registered higher antioxidant activity (FRAP assay) and a more significant difference (*p* < 0.05) than Manuka honey (648.25 75 μM Fe (II)/100 g) [90]. A similar case was observed in the TPC of Thai honeys, with higher values for mangosteen (1495.79 mg GAE/kg) and rambutan (1361.68 mg GAE/kg) than Manuka honey (657.91 mg GAE/kg). Significant differences between their antioxidant activity values (DPPH) were reported. While only higher values for antioxidant activity (FRAP) were observed for mangosteen honey [110].

However, honeys of the same botanical origin have presented different values of TPC and TFC (Table 1), because their presence depends on their pollen pattern, which in turn can be influenced by geographical factors such as climate and altitude, among others. The geographical origin also influences the total phenol compound content in the honeys. Chilean honeys could be easily differentiated from others, showing that samples from the same geographical region share similar characteristics in the content of phenol and flavonoid compounds [99]. Honeys produced in Germany reported differences in TPC between Acacia honey(627.56 ± 44.03 mg GAE/100 g) and the wild carrot from Argelia (503.09 ± 8.29 mg GAE/100 g) [111]. Monofloral honeys from China of the same floral origin, but of different geographical origin, showed significant differences in TPC values, except in Acacia and Coptidis samples [49]. In multiflorals from Nepal, a significant difference (*p* < 0.01) was observed in the mean value of TPC, according to the different altitudes at which they were collected. The honeys collected at low altitudes of 800–1500 m had a higher phenol content (61.77 mg GAE/100 g) [112], in comparison to those harvested at high altitudes of 1500–3500 m, whose value was of 118.65 mg GAE/100 g. This is attributed to the fact that the plants cultivated at high altitudes synthesize secondary antioxidant metabolites in order to protect themselves from the extreme climatic conditions.

The climate also has an influence on the quantity of phenolic compounds. In honeys from Kenya, significant differences (*p* < 0.05) were reported in total phenol content, in those produced in a region with high precipitation (141.7177 mg GAE/100 g) and those collected in a hot and humid climate (116.1777 mg GAE/100 g), as well as in semiarid one (98.3777 mg GAE/100 g) [92]. The same differences were observed in the total flavonoid content, but there were similarities among the honeys produced in a hot and humid climate (35.47 mg QE/100 g) and fresh and humid climate (29.19 mg QE/100 g). These results could be linked to the differences in vegetation and availability of the melliferous plants due to the climate.

Determining the phenol profile allows us to observe the presence of certain compounds in honey with a specific botanical origin. The phenolic acids and flavonoids present in the honey can be used as differentiators of floral origin. Some phenolic compounds are indispensable for the functioning of plants; others are useful for defense mechanisms in stressful situations [84]. Honeydew (conifers) have been found to contain protocatechuic acid at significant concentrations, observed in diverse studies. It has been suggested that protocatechuic acid can be found in the honey of the coniferous trees of Greece and Turkey and distinguishes them from those that are multifloral [25]. In Turkish honeydews, this compound was detected in all samples (pine, oak, quercus), while ellagic acid was only detected in significant amounts in oak and quercus honeydew [113]. However, other studies have observed certain phenolic compounds regardless of the botanical origin of the honey. Methyl syringate, considered a floral marker of Manuka honey, but has been detected in Tunisian multifloral samples [113]. Naringenin has been suggested as a marker of the botanical origin of honey with *M. caesalpiniifolia* pollen type (Fabaceae/Mimosoideae) [114]. Ulmo honey from Chile present Abscisic acid is presents abscisic acid in a higher concentration compared to other phenolic compounds, so it could be considered as its floral marker [115]. In Italian monoflorals certain compounds that could be associated with their floral origin were observed: myricetin for *Taraxacum* sp. honey; apigenin, caffeic acid, quercetin and p-coumaric acid for *C. sativa* honey; caffeic acid and chlorogenic acid for *Erica* sp. honey; quercetin, chlorogenic acid and p-coumaric acid for honeydew. However, the authors emphasize that the phenolic profile alone cannot define the botanical origin of honey [58]. This is because honey is a very complex natural product.

The analysis of phenolic compounds can be a complementary method to determine the botanical origin of honey, since there are not enough studies that prove a close correlation between them. For honeys from Greece, it was shown that phenolic compounds in combination with conventional quality parameters and chemometrics may be able to differentiate the geographical origin, but not by themselves [116]. In Argentine honeys of eucalyptus, trefoils, and alfalfa, it was not possible to identify phenolic compounds as biomarkers of floral origin [117]. Furthermore, with the analysis of phenolic compounds, it is not possible to identify adulterations such as the addition of pollen to honey or its secondary species. Therefore, it is necessary to use it as a complementary alternative for the identification and authentication of the botanical origin. However, phenolic compounds could be considered as a mandatory tool for botanical and geographical discrimination of honey, in order to protect against possible fraud.

## 5. The Color of the Honey and Phenolic Compounds

The analytical methods, the sensory evaluation of honey can complement the confirmation of the botanical origin, establish sensory profiles for monofloral honey, verify quality, among others [118], since various studies have observed differences in the characteristics of honeys according to their floral and geographical origin [50,60,119,120,121]

Despite this, the botanical origin of honeys is one of the factors that determines their sensory characteristics. For example, alfalfa honeys are characterized by a very light color and weak odor, flavor and persistence. On the other hand, Eucalyptus honeys are characterized by having an intense aroma and flavor and crystallize, forming small crystals. The honeys trefoils have a clear, delicate odor and flavor, and the appearance of medium crystals [117]. Sensory differences were found in stingless bee honeys (*Plebeia molesta*) according to their environment and different geographical origin. Honeys from the coast of Salinas showed great fluidity, while the honeys from Forest Serrano were characterized by their amount of crystals; finally, those from Plain Forest were distinguished by their persistence of taste [122]. However, different sensory characteristics have been reported between honeys from the same floral source, which means that their properties and composition not only depend on the sources that provide the pollen, but also on other factors, such as geographical origin, harvesting and storage conditions [60] This subject is very complex coarse, so in this review, we will only focus on the color of honey as the sensory characteristic in relation to the content of phenolic compounds.

The color of the honey is a parameter that indicates the presence of pigments; it is influenced by its botanical origin, the composition of the nectar, the process of acquisition, temperature, and storage time. The colors range from a nearly colorless white to a dark red, passing through yellow and amber tones [4].

The changes in color could be due to interventions of the beekeeper, or different production and conservation methods, such as, the use of old panels, contact with metals or exposure to high temperatures and light [41]. Color is the physical property that is immediately observed by the consumer and can be influential at the moment of purchase [123]. Delmoro et al. [124] reported that, in North America, consumers preferred lighter honeys and of a whitish tone, because they have a less intense flavor, while in Europe, the consumers prefer darker honeys, with amber tones and a more intense flavor. The determination of the color of honey is carried out by methods that measure reflectance or transmittance, using spectrophotometers or tristimulus colorimeters. The most-used methods, according to the studies reported, are the Pfund colorimetric method and the CIELab chromatic analysis.

The internationally standardized method is the Pfund method, where the value of absorbance is converted to mm, which range from 0 to 114 mm and are named according to the standard nomenclature, which range from “water-white” to “dark amber” [124]. In various studies, where this methodology has been applied, it was observed that the color of honey will vary according to its botanical origin. In multifloral, there is a greater variation because they are produced from different nectars, while the monofloral honeys present a defined color (Table 2). The limiting factor in the use of this method is that the color will be classified according to the established scale; however, it does not detect the small differences in a particular color for each sample.

The reflectance methods determine the tristimulus values L, a and b, Chroma and Hue [129]. The value L* estimates the degree of luminosity, positive values for a* indicate red, negative a* values indicate green, positive b* values indicate yellow and negative b* values indicate blue [76]. In various studies, positive b* values have been reported and thus, the honeys present tones of yellow (Table 3); however, in some other studies, greater values have been reported for a* in comparison with b* values, which indicate the appearance of red tones, as in the case of lavender honey from Spain, lychee honey from China and orange blossom from Mexico [44]. An interesting case is the Chinese honey *Amorpha fruticosa*, whose value of a* (109.03) reflects a pink or red tone and is the highest value that has been reported until now [76]. In Estonian honeys, through a correlation coefficient between L* and a* value, they verified that the darker the honey, the more reddish tones it presents [60].

The intensity of the color is represented by ABS450, where the absorbance is measured with two wavelengths (450 and 720 nm) and the difference between the absorbance values was reported in milli absorbance units (mAU) [132]. ABS450 is a measurement that confirms the presence of pigments such carotenoids, minerals, pollen, phenolics, and flavonoids, which are known to exhibit antioxidant properties [41,133]. In various studies, this method was utilized to deduce high concentrations of phenolic compounds and flavonoids or antioxidant properties of honey (Table 4). However, more research is needed to assert this correlation.

Various studies have linked the color of the honeys to the phenolic compound content and its antioxidant capacity. Islam et al. [133] reported a high correlation between color intensity (ABS_450_) and antioxidant activity, determined by DPPH (*r* = 0.984; *p* = 0.01) and FRAP (*r* = 0.914; *p* = 0.01) in monofloral and multifloral honey from Bangladesh. Kavanagh et al. [95] reported a positive correlation between the color (Pfund) of rural and urban honeys and TPC (*r* = 0.6; *p* < 0.001).

Cabrera et al. [66] grouped honeys from the humid region of Chaco, in Argentina, according to their color (extra light amber, amber and dark amber), and reported a higher quantity of phenols and flavonoids in darker samples. The color intensity of amber was attributed to flavonoid content, since a better correlation was observed (*r* = 0.78) than with total phenolic compound content (*r* = 0.53). In Argentina, Ciappini et al. [123] reported that the multifloral honeys of the region possess darker tones than the monofloral variety, presenting a positive correlation (*r* = 0.93) between flavonoid content and color. Al-Farsi et al. [89] observed that the dark amber honeys, with the highest values on the Pfund scale (227 nm), also exhibited the highest flavonoid (2145 mg catechin/kg) and phenolic compound (2336 mg GAE/kg) values. Additionally, they reported a strong correlation between color and flavonoid (*r =* 0.999) and phenolic compound (*r =* 0.974) content. In Mexico, Balcázar-Cruz et al. [129] found a positive correlation (*r* = 0.864) between the values of color and flavonoid content, concluding that, as the honey got darker, the flavonoid content increased.

In contrast, Pontis et al. [128] reported higher values for phenol content (509–548 mg GAE/kg), than for flavonoids (47.1–48.6 mg QE/kg) for the darkest samples (151.08–166.68 mm Pfund) of multifloral Brazilian honeys. Daci-Ajvazi et al. [134] indicate that those Kosovo honeys of dark brown color exhibited a higher phenol content compared to others of a light-yellow tone, demonstrating a positive correlation between color intensity (ABS_450_) and phenol content (*r* = 0.711; *p* = 0.001).

Jasicka-Misiak et al. [7] observed that the color intensity of the honeys is lower in fresh samples, increasing in the oldest samples; this is probably due to the reactions that take place during storage. The dark tones of honey can also be due to the formation of Maillard reaction products as temperature increases. Those with high quantities of free amino groups and reducing sugars presented greater darkening thanks to these products [44]. Regular and prolonged heat increases the quantity of phenolic compounds and antioxidant activity without specifying the factor of this growth [94]. However, there are few studies which report the influence of the storage conditions in the development of the color of honey.

## 6. Conclusions

Phenolic compounds are one of the most widely distributed secondary metabolites in nature. The study of foods that contain phenolic compounds has been of great relevance in recent years, due largely to the beneficial effects on human health. Its presence in honey confers it biological and functional activity, besides providing biomarkers that allow the determination of the floral origin and some sensory characteristics.

The total phenol and flavonoid content have become an indispensable assay for the determination of the quality of a honey, for its nutritional and sensory value, among others. The phenols and flavonoids commonly reported in various studies are p-coumaric acid, caffeic acid, chlorogenic acid, rutin, myricetin, and apigenin. The most effective method for the determination of the phenol and flavonoid profile present in honeys is still being evaluated. Moreover, various studies have reported a higher positive correlation between total phenol content and the antioxidant activity of honey, while the color of honey has a stronger correlation with the total flavonoid content.

The darkest honey presents high total flavonoid content values. The variations in color are frequently present in multifloral, in accordance with the differences in the percentages of nectar used for its production. Occasionally, said variability in its composition gives higher phenolic compound values; however, monofloral honeys with a higher phenol and flavonoid content have also been found.

There is a direct relationship between the botanical-geographical origin and the sensory and biological characteristics of the honeys, which define its identity and link with the local production and territory [127]. This relationship exhibits a growing interest in ensuring the quality of the honeys by obtaining differentiated quality, such as Protected Designation of Origin, which gives an added value to the product, both at national and international levels [135]. From this perspective, an opportunity arises for researchers to link the phenolic compounds, antioxidant activity and other characteristics of the honey, not only with the floral origin, but with the geographical, production, and territorial origins.

## Figures and Tables

**Table 1:** Total Phenol Content (TPC) and Total Flavonoid Content (TFC) in honeys according to botanical.

Botanical Origin	Total Phenolic Contents (TPC)	Total Flavonoid Contents (TFC)	Country/Region	Reference
Acacia (*Acacia mangium*)	129.16–341.67 mg GAE/kg	28.83–113.06 mg QE/kg	Malasya	[41]
	33.12–55.86 mg GAE/100 g	2.96–5.81 mg QE/100 g	Malasya/Johor	[42]
	33.21 mg GAE/100 g	2.71 mg QE/100 g	Iran	[43]
	24.3 mg GAE/100 g	1.48 mg CAE/100 g	Hungary	[44]
	16.5 mg GAE/100 g	4.15 mg QE/100 g	European countries	[45]
	52.7–36.57 mg GAE/100 g	n.d.	China	[46]
	0.51–0.63 mg GAE/g	n.d.	Korea	[47]
	0.02 mg GAE/g	n.d.	Turkey/Altınordu	[48]
	10.43–12.54 mg PCE/100 g	9.14–12.17 mg QE/100 g	China	[49]
Asteraceae	49.6 mg GAE/100 g	3.3 mg QE/100 g	Algeria/Babors Kabylia	[50]
Black locust (*Robinia pseudoacacia* L.)	28.2–52.0 mg GAE/kg	n.d.	Croatia	[51]
Blueberry	79.2 mg GAE/100 g	8.4 mg CAE/100 g	Canada	[44]
Buckwheat (*Fagopyrum esculentum*)	922.52–1876.58 mg GAE/kg	n.d.	Poland/Podkarpackie	[52]
	105.88–1267.94 mg GAE/100 g	n.d.	Poland	[53]
	211.0 mg GAE/100 g	96.6 mg CAE/100 g	Poland	[54]
	55.0 mg GAE/100 g	47.0 QE/100 g	Republic of Moldova/Balti	[55]
Carob (*Prosopis pallida*)	121.81 mg GAE/100 g	1.0063 mg QE/100 g	Peru	[56]
Chestnut (*Castanea* sp.)	1.83 mg GAE/g	n.d.	Korea/Yangyang	[47]
	0.12 mg GAE/g	n.d.	Turkey/Gurgentepe	[48]
	487–1134 mg GAE/kg	n.d.	Portugal	[57]
	129.2–212.7 mg GAE/kg	n.d.	Croatia	[51]
Citrus	14 mg GAE/kg	n.d.	Greece	[25]
	167.8 mg GAE/kg	60.8 mg QE/kg	Italy	[58]
	83.85 mg GAE/100 g	69.53 µg QE/100 g	India/Sohra	[59]
Coffee	85.2 mg GAE/100 g	8.30 mg CAE/100 g	Guatemala	[44]
Coriander	68.7 mg GAE/100 g	8.02 mg QE/100 g	Iran	[43]
Dandelion (*Taraxacum officinale*)	326.13–738.74 mg GAE/kg	n.d.	Poland/Podkarpackie	[52]
	256.7 mg GAE/kg	95.7 mg QE/kg	Italy	[58]
	33.73–139.15 mg GAE/100 g	n.d.	Poland	[53]
Eucalyptus (*Eucalyptus* sp.)	150.08–180.37 mg GAE/100 g	1.4058–1.6264 mg QE/100 g	Peru	[56]
	957.0 mg GAE/kg	n.d.	Australia	[57]
	320.0 mg GAE/kg	165.4 mg QE/kg	Italy	[58]
	120.6–134.0 mg PCE/100 g	73.51–102.1 mg QE/100 g	China	[49]
Fir	20–52 mg GAE/kg	n.d.	Greece	[25]
Forest	23 mg GAE/100 g	11.4 mg QE/100 g	European countries	[45]
	24 mg GAE/kg	n.d.	Greece	[25]
Gelam (*Melaleuca cajuputi*)	27.33–52.25 mg GAE/100 g	2.38–6.53 mg QE/100 g	Malasya/Terengganu	[42]
Genista	52.6 mg GAE/100 g	3.2 mg QE/100 g	Algeria/Babors Kabylia	[50]
Goldenrod (*Solidago virgaurea*)	284.68–966.67 mg GAE/kg	n.d.	Poland/Podkarpackie	[52]
	351.87 mg GAE/100 g	n.d.	Poland	[53]
Heather	109.8 mg GAE/100 g	7.9 mg QE/100 g	Algeria/Babors Kabylia	[50]
	1224 mg GAE/kg	n.d.	Portugal	[57]
	88.7 mg GAE/100 g	6.4 mg QE/100 g	Estonia	[60]
Honeydew	1059–1910 mg GAE/kg	n.d.	Portugal	[57]
	372.97–1001.02 mg GAE/kg	n.d.	Poland/Podkarpackie	[52]
	640.0 mg GAE/kg	n.d.	Greece	[57]
	147.8–588.0 mg GAE/kg	n.d.	Croatia	[51]
	110.41–897.34 mg GAE/100 g	n.d.	Poland	[53]
	201.0 mg GAE/100 g	121.3 mg CAE/100 g	Poland	[54]
	128.3 mg GAE/100 g	8.7 mg QE/100 g	Algeria/Babors Kabylia	[50]
	50.04–243.86 mg GAE/100 g	1.81–25.22 mg CAE/100 g	Spain	[61]
	65.67 mg GAE/kg	10.18 mg CAE/kg	Malasya/Gertak Sanggul	[62]
	11–50 mg GAE/kg	n.d.	Greece	[25]
Jambul Merak	91.32 mg GAE/kg	10.27 mg CAE/kg	Malasya/Padang Terap	[62]
	114.38 mg GAE/kg	12.68 mg CAE/kg	Malasya/Kubang Pasu	[62]
Jujube (*Ziziphus* sp.)	190.79 mg GAE/100 g	16.43 mg QE/100 g	Iran	[43]
	174.9 mg GAE/100 g	3.4 mg QE/100 g	Algeria	[63]
Jujube (*Ziziphus* sp.)	114.63–136.17 mg GAE/100 g	n.d.	China	[46]
	362–618 mg PCE/kg	n.d.	China	[64]
Korean raisin	1.43 mg GAE/g	n.d.	Korea/Yangyang	[47]
Lavender	85.6 mg GAE/100 g	11.2 mg CAE/100 g	Spain	[44]
Linden	133.1 mg GAE/100 g	61.2 mg CAE/100 g	Poland	[54]
	54.54–117.84 mg GAE/100 g	n.d.	Poland	[53]
	12.30–15.03 mg GAE/100 g	n.d.	China	[46]
	128.1–147.3 mg PCE/100 g	20.92–30.32 mg QE/100 g	China	[49]
Litchi	54.6 mg GAE/100 g	6.38 mg CAE/100 g	China	[44]
Manuka (*Leptospermum scorparium*)	1.18 mg GAE/g	n.d.	New Zealand	[47]
	203.5–217.0 mg GAE/100 g	118.9–115.7 mg CAE/100 g	New Zealand	[54]
Meadow	21.3 mg GAE/100 g	6.14 mg QE/100 g	European countries	[45]
Multifloral	236.94–1021.62 mg GAE/kg	n.d.	Poland/Podkarpackie	[52]
	744–1277 mg GAE/kg	n.d.	Portugal	[57]
	1199 mg GAE/kg	n.d.	Greece	[57]
	170.0 mg GAE/100 g	n.d.	Mexico	[65]
	141.0 mg GAE/100 g	56.8 mg CAE/100 g	Poland	[54]
	81.22–983.04 mg GAE/100 g	n.d.	Poland	[53]
	140.83 mg GAE/100 g	144.84 µg QE/100 g	India/Shillong	[59]
	126.07 mg GAE/100 g	72.49 µg QE/100 g	India/Mawsynram	[59]
	74.42 mg GAE/100 g	25.49 µg QE/100 g	India/Tezpur	[59]
	40.18–118.82 mg GAE/100 g	6.94–67.76 mg QE/100 g	Argentine	[66]
	60.5 mg GAE/100 g	4.1 mg QE/100 g	Algeria/Babors Kabylia	[50]
	26.2–68.6 mg GAE/100 g	1.9–5.7 mg QE/100 g	Estonia	[60]
	0.26 mg GAE/g	n.d.	Turkey/Mesudiye	[48]
Orange	49.6 mg GAE/100 g	5.05 mg CAE/100 g	México	[44]
	104.0 mg GAE/kg	n.d.	Greece	[57]
Piura honey	145.28 mg GAE/100 g	3.8391 mg QE/100 g	Peru	[56]
Rape (*Brassica* sp.)	205.41–310.81 mg GAE/kg	n.d.	Poland/Podkarpackie	[52]
	58.41–223.72 mg GAE/100 g	n.d.	Poland	[53]
	296.68 mg GAE/100 g	155.26 µg QE/100 g	India/Jorhat	[59]
Rape (*Brassica* sp.)	8.70–1.20 mg GAE/100 g	n.d.	China	[46]
Rubber Tree	74.11 mg GAE/kg	11.77 mg QE/kg	Malasya/Jasin	[62]
Rubus	53.9 mg GAE/100 g	3.6 mg QE/100 g	Algeria/Babors Kabylia	[50]
Starfruit (*Averrhoa carambola* L)	39.38–40.44 mg GAE/100 g	8.46–9.31 mg QE/100 g	Malasya/Pahang	[42]
Sulla (*Hedysarum*)	52.6 mg GAE/100 g	3.8 mg QE/100 g	Algeria/Babors Kabylia	[50]
Sunflower	22.1 mg GAE/100 g	10.9 mg QE/100 g	European countries	[45]
Thyme (*Thymus* sp.)	953.0 mg GAE/kg	n.d.	Greece	[57]
	1901.0 mg GAE/kg	n.d.	New Zealand	[57]
	250.0 mg GAE/kg	83.5 mg QE/kg	Italy	[58]
	17–24 mg GAE/kg	n.d.	Greece	[25]
	76.85 mg GAE/100 g	6.09 mg QE/100 g	Iran	[43]
Tilia (*Tilia* sp.)	302.70–549.55 mg GAE/kg	n.d.	Poland/Podkarpackie	[52]
	260.0 mg GAE/kg	55.0 mg QE/kg	Italy	[58]
	66.2–121.0 mg GAE/kg	n.d.	Croatia	[51]
	0.5 mg GAE/g	n.d.	Korea/Yangyang	[47]
Wild Honey	150.91–207.89 mg GAE/100 g	1.432–1.7563 mg QE/100 g	Peru	[56]
Zapote (*Capparis angulata*)	134.87–143.72 mg GAE/100 g	0.9139–0.9651 mg QE/100 g	Peru	[56]

GAE: gallic acid equivalents; PCE: protocatechuic acid equivalents; QE: quercetin equivalents; CAE: catechin equivalents; n.d. not determined.

**Table 2:** Pfund values for monofloral and multifloral honeys.

Botanical Origin	Pfund (mm)	Colour	Type	Region/Country	Reference
Acacia honey	129.8–336.2	Dark amber	monofloral	Oman	[89]
Baiker (*Jusdticia adhatoda*)	27.95	White	monofloral	Pakistan	[125]
Bari (*Ziziphus jojoba*)	78	Light amber	monofloral	Pakistan	[125]
Berseem (*Trifolium repens*)	47.38	Extra light amber	monofloral	Pakistan	[125]
Clover	29.7	White	monofloral	Pampean, Argentine	[123]
Del Delta honey	40–140	Extra light amber-Dark amber	multifloral	Delta del río Paraná/Argentine	[123]
Eucalyptus	31	White	monofloral	Blida/Algeria	[126]
	58.1	Light amber	monofloral	Pampean/Argentine	[123]
*Euphorbia dendroides*	105	Amber	monofloral	El Bayadh/Algeria	[126]
*Helianthemum*	27	white	monofloral	Mostaganem/Algeria	[126]
Humid Chaco honey	40.67–140	Extra light amber-dark amber	multifloral	Argentine	[66]
Orange (*Citrus x sinensis*)	47	Extra light amber	monofloral	Pakistan	[125]
Ortigueira honey	50–85	White, Light amber, Amber	multifloral	Brazil	[127]
Phulai (*Acacia modesta*)	52.67	Light amber	monofloral	Pakistan	[125]
Roraima honey	35.58–166.68	Extra light amber-Dark amber	multifloral	Brazil	[128]
*Schefflera abyssinica*	53.1	Extra light amber	monofloral	Ethiopia	[91]
Serson (*Brassica campestris*)	40.5	Extra light amber	monofloral	Pakistan	[125]
Sheesham (*Dalbergia sisso*)	48	Extra light amber	monofloral	Pakistan	[125]
Sidr honey	65.5–180.5	Amber-dark amber	multifloral	Oman	[89]
Tabasco honey	34–85	Extra light amber-Light amber	multifloral	México	[129]
Ziziphus honey	48	Extra light amber	monofloral	Laghouat/Algeria	[126]
*Zygophyllum album*	198	Dark amber	monofloral	El Oued/Algeria	[126]

**Table 3:** L*, a*, b* values for monofloral and multifloral honey.

Botanical Origin	L*	a*	b*	Type	Country/Region	Reference
Acacia	11.97	5.19	24.18	monofloral	Hungary	[44]
Alfalfa	31.03	2.94	24.85	monofloral	Iran/Baharestan	[130]
*Amorpha fruticosa*	29.67	109.03	4.37	monofloral	China	[76]
Astragal	30.12	2.63	23.91	monofloral	Iran/Shahr-e- Kord	[130]
Blueberry	5.65	10.57	6.3	monofloral	Canada	[44]
Borneo (*Acacia mangium*)	25.86	1.45	2.66	multifloral	Malaysia/Kudat	[131]
Coffee	3.32	1.55	1.77	monofloral	Guatemala	[44]
Coriander	18.98	23.01	31.64	monofloral	Iran/Tiran and Karvan	[130]
Dill	29.94	18.55	47.68	monofloral	Iran/Tiran and Karvan	[130]
Gelam (*Melaleuca cajuputi*)	26.06	2.1	2.78	monofloral	Malaysia/Merchang	[131]
Kelulut (*Acacia mangium*)	24.9	1.9	2.52	multifloral	Malaysia/Teluk Intan	[131]
Lavender	4.83	5.96	4.91	monofloral	Spain	[44]
Litchi	4.2	3.85	3.08	monofloral	China	[44]
Orange blossom	3.58	4.24	3.09	monofloral	México	[44]
	33.1	2.77	23.22	monofloral	Iran/Shiraz	[130]
Ortigueira	45.46–56.33	0.43–2.46	24.78–30.9	multifloral	Brazil	[127]
Parsley	45.63	5.14	36.5	monofloral	Iran/Tiran and Karvan	[130]
Pineapple (*Ananas comosus*)	27.31	2.05	3.59	monofloral	Malaysia/Rengit	[131]
Qanqal	45.05	5.41	37.67	monofloral	Iran/Shahr-e- Kord	[130]
Tamarisk	32.47	2.27	21.8	monofloral	Iran/Shahr-e- Kord	[130]
Thyme	41.39	11.34	49.81	monofloral	Iran/Damavand	[130]
Tualang (*Koompassia excelsa*)	26.52	1.42	2.96	monofloral	Malaysia/Tasik Pedu	[131]
Ziziphus	41.19	14.29	59.98	monofloral	Iran/Borazjan	[130]

**Table 4:** ABS_450_ values for honeys of different floral origin.

Botanical Origin	ABS_450_ (mAU)	Type	Country/Region	Reference
*Astragalus* spp.	129.26	monofloral	Iran	[132]
*Astragalus adscendens*	53.3	monofloral	Iran	[132]
Mustard (*Brassica campestris*)	843.0	monofloral	Bangladesh	[133]
Buckwheat	>1.4	monofloral	Poland	[52]
*Eucalyptus* spp.	104.73	monofloral	Iran	[132]
Goldenrod	<0.8	monofloral	Poland	[52]
Ramtil (*Guizotia abyssinica*)	2021.67	monofloral	Bangladesh	[133]
*Helianthus annuus*	180.82	monofloral	Iran	[132]
Honeydew (*Gertak Sanggul*)	512.0	monofloral	Malaysia/Penang	[62]
Jambul Merak (*Kubang Pasu*)	1141.33	monofloral	Malaysia/Kedah	[62]
Jambul Merak (*Padang Terap*)	950.0	monofloral	Malaysia/Kedah	[62]
Litchi (*Litchi chinensis*)	254.0	monofloral	Bangladesh	[133]
Mixed source	785.33–2034.0	multifloral	Bangladesh	[133]
Black seed or kali jeera (*Nigella sativa*)	9178.33	monofloral	Bangladesh	[133]
Rape	<0.4	monofloral	Poland	[52]
Rubber Tree (Jasin)	778.33	monofloral	Malaysia/Melaka	[62]
Til *(Sesamum indicum)*	1217.67	monofloral	Bangladesh	[133]
*Thymus vulgaris*	165.88	monofloral	Iran	[132]
Tile	<0.8	monofloral	Poland	[52]
*Ziziphus spina-christi*	250.99	monofloral	Iran	[132]

ABS_450_: color intensity mAU: milli absorbance units.

## Supplementary Materials

The following are available online at https://www.mdpi.com/article/10.3390/antiox10111700/s1, Table S1: Phenolic acids in honeys of different floral origin, Table S2: Flavonoid acids in honeys of different floral origin.
