# Supplementary material for: Phenolic Compounds in Honey and Their Relationship with Antioxidant Activity, Botanical Origin, and Color
Source: Antioxidants (Basel). 2021 Oct 27;10(11):1700. doi: 10.3390/antiox10111700 (PMC8614671; doi:10.3390/antiox10111700)
Supplement: Supplementary file 1 [file antioxidants-10-01700-s001.zip › antioxidants-1385734-supplementary.pdf]

Table S1. Phenolic acids in honeys of different floral origin

Botanical origin	Phenolic acids														Country	Reference
	p-Coumaric acid	Caffeic acid	Protocatechuic acid	vanillic acid	4-Hidroxybenzoic acid	Syringic acid	Chlorogenic acid	Ferulic acid	Gallic acid	Benzoic acid	Cinnamic acid	2,4 Dihydroxybenzoic acid	Ellagic acid	Rosmarinic acid		
Bracatinga (Mimosa scabrella Bentham) μg/100g	26.53 – 57.18	5.612 – 51.89	n.d.	n.d.	n.d.	17.32 – 78.91	12.55 – 89.31	16.16 – 57.16	22.22 – 40.29	116.4 –1048	n.d.	117.7 – 195.1	n.d.	n.d.	Brazil	[70]
Safflower *(Carthamus tinctorius)* g/kg	n.d.	0.04	0.12	3.19	1.524	n.d.	n.d.	0.25	0.636	n.d.	0.56	n.d.	n.d.	n.d.	China	[71]
Fir μg/kg	219–514	377–254	3258–16777	149–307	1121–1438	n.d.	n.d.	n.d.	n.d.	n.d.	n.d.	n.d.	n.d.	n.d.	Greece	[25]
Thyme μg/kg	143–252	113–134	300–649	103–245	1070–1724	n.d.	n.d.	n.d.	n.d.	n.d.	n.d.	n.d.	n.d.	n.d.		
Amorpha fruticosa g/kg	1.46	0.26	n.d.	n.d.	n.d.	36.43	n.d.	1.89	1.13	n.d.	n.d.	n.d.	n.d.	n.d.	China	[72]
Forest Fino μg/kg	288	445	2394	237	1503	n.d.	n.d.	n.d.	n.d.	n.d.	n.d.	n.d.	n.d.	n.d.	Greece	[25]
Pine μg/kg	211–701	376–741	3058–5967	340–376	1460–4059	n.d.	n.d.	n.d.	n.d.	n.d.	n.d.	n.d.	n.d.	n.d.		
Citrus μg/kg	135	92	303	71	889	n.d.	n.d.	n.d.	n.d.	n.d.	n.d.	n.d.	n.d.	n.d.		
Pine honeydew (*Pinus sylvestris L.*) mg/100g	n.d.	0.049 – 0.337	n.d.	n.d.	n.d.	n.d.	0.214 – 0.824	0.039 – 0.620	1.315 – 4.793	n.d.	n.d.	n.d.	n.d.	n.d.	Poland	[69]

Table S1. *Cont.*

Botanical origin	Phenolic acids														Country	Reference
	**p-Coumaric acid**	**Caffeic acid**	**Protocatechuic acid**	**vanillic acid**	**4-Hidroxybenzoic acid**	**Syringic acid**	**Chlorogenic acid**	**Ferulic acid**	**Gallic acid**	**Benzoic acid**	**Cinnamic acid**	**2,4 Dihydroxybenzoic acid**	**Ellagic acid**	**Rosmarinic acid**		
Ulmo honey (*Eucryphia cordifolia*) mg/100g	0.14–0.41	0.14–0.44	n.d.	n.d.	n.d.	n.d.	0.15–0.21	n.d.	0.24–0.36	n.d.	n.d.	n.d.	n.d.	n.d.	Chile	[73]
Willow (*Salix spp.*) mg/100g	n.d.	0.070 – 0.159	n.d.	n.d. n.d.	0.717 – 1.323	n.d.	0.301 – 0.493	0.091 – 0.486	n.d.	n.d.	n.d.	n.d.	n.d.	n.d.	Poland	[69]
Carob (*Prosopis pallida*) mg/kg	1.572	n.d.	n.d.	n.d.	n.d.	0.722	0.029	n.d.	n.d.	n.d.	n.d.	n.d.	n.d.	n.d.		
Eucalyptus (*Eucalyptus globulus*) mg/kg	0.11–0.797	n.d.	n.d.	n.d.	n.d.	0.037 – 0.301	0.007 – 0.144	n.d.	n.d.	n.d.	n.d.	n.d.	n.d.	n.d.	Peru	[54]
Wild Honey mg/kg	0.010 – 0.925	n.d.	n.d.	n.d.	n.d.	0.018 – 0.340	0.009 – 0.041	n.d.	n.d.	n.d.	n.d.	n.d.	n.d.	n.d.		
Heather (*Calluna vulgaris L.*) mg/100g	0.354 – 0.690	0.142 – 0.316	n.d.	n.d.	n.d.	n.d.	0.113 – 0.172	n.d.	n.d.	n.d.	n.d.	n.d.	n.d.	n.d.	Poland	[69]
Manuka (*Leptospermum scoparium*) mg/100g	2.879 – 2.938	n.d	n.d.	n.d.	n.d.	n.d.	n.d.	n.d.	n.d.	n.d.	n.d.	n.d.	0.519 – 0.525	0.262 – 0.291	New Zealand-Australia	[69]
Buckwheat (*Fagopyrum esculentum L.*) mg/100g	0.267 – 0.489	n.d.	n.d.	n.d.	n.d.	n.d.	n.d.	n.d.	n.d.	n.d.	n.d.	n.d.	n.d.	n.d.	Poland	[69]

n.d. not determined

Table S2. Flavonoid acids in honeys of different floral origin

Botanical Origin	Flavonoids acids													Country	Reference
	**Rutin**	**Myricetin**	**Apigenin**	**Quercetin**	**Quercetin 3-O-glucoside**	**Naringenin**	**Isorhamnetin**	**Luteolin**	**Pinocembrin**	**Kaempferol**	**Gallocatechin gallate**	**Hesperidin**	**Pinobanksin**		
Bracatinga *(Mimosa scabrella Bentham)* µg/100g	9.190 – 39.89	n.d.	0.502 – 4.670	n.d.	n.d.	1.731 – 10.66	8.02–11.72	4.5–16.42	1.550 – 3.321	14.77 – 34.44	n.d.	5.70–98.85	n.d.	Brazil	[70]
Carthamus tinctorius L. g/kg	0.39	1.02	n.d.	5.34	0.3	0.07	n.d.	n.d.	n.d.	0.13	n.d.	n.d.	0.38	China	[71]
Buckwheat honey µg/kg	19.44	n.d.	18.76	1.1	n.d.	n.d.	131.17	17.39	n.d.	n.d.	594.46	n.d.	n.d.	Republic of Moldova	[53]
Fagopyrum esculentum Moench.	0.019	n.d.	n.d.	0.001	n.d.	n.d.	0.131	n.d.	n.d.	n.d.	0.594	n.d.	n.d.		
Amorpha fruticosa L. g/kg	2.21	n.d.	n.d.	0.27	n.d.	0.03	n.d.	0.12	0.12	n.d.	n.d.	n.d.	n.d.	China	[72]
Ulmo honey (*Eucryphia cordifolia*) mg/100g	n.d.	n.d.	0.01–0.03	0.17–0.26	n.d.	n.d.	n.d.	0.05–0.10	0.61–2.73	n.d.	n.d.	n.d.	n.d.	Chile	[73]
Wild Honey mg/kg	0.008 – 0.121	n.d.	0.346 – 0.652	n.d.	n.d.	n.d.	n.d.	n.d.	n.d.	n.d.	n.d.	n.d.	n.d.	Peru	[54]
Carob (*Prosopis pallida*) mg/kg	0.073	n.d.	0.191	n.d.	n.d.	n.d.	n.d.	n.d.	n.d.	n.d.	n.d.	n.d.	n.d.		
Eucalyptus (*Eucalyptus globulus*) mg/kg	0.006 – 0.024	n.d.	0.341 – 0.652	n.d.	n.d.	n.d.	n.d.	n.d.	n.d.	n.d.	n.d.	n.d.	n.d.		
Buckwheat (*Fagopyrum esculentum L.*) mg/100g	n.d.	0.243 – 0.606	n.d.	n.d.	n.d.	n.d.	n.d.	n.d.	n.d.	n.d.	n.d.	n.d.	n.d.	Poland	[69]
Heather (*Calluna vulgaris L.*) mg/100g	n.d.	0.639 – 0.861	n.d.	n.d.	n.d.	n.d.	n.d.	n.d.	n.d.	n.d.	n.d.	n.d.	n.d.		

Table S2. *Cont.*

Flavonoids acids															
**Botanical Origin**	**Rutin**	**Myricetin**	**Apigenin**	**Quercetin**	**Quercetin 3-O-glucoside**	**Naringenin**	**Isorhamnetin**	**Luteolin**	**Pinocembrin**	**Kaempferol**	**Gallocatechin gallate**	**Hesperidin**	**Pinobanksin**	**Country**	**Reference**
Pine honeydew (*Pinus sylvestris L.*) mg/100g	n.d.	0.179 – 0.824	n.d.	n.d.	n.d.	n.d.	n.d.	n.d.	n.d.	n.d.	n.d.	n.d.	n.d.		
Willow (*Salix spp.*) mg/100g	n.d.	0.172 – 0.641	n.d.	n.d.	n.d.	n.d.	n.d.	n.d.	n.d.	n.d.	n.d.	n.d.	n.d.		
Manuka (*Leptospermum scoparium*) mg/100g	n.d.	1.052 – 1.261	n.d.	n.d.	n.d.	n.d.	n.d.	n.d.	n.d.	n.d.	n.d.	n.d.	n.d.	New Zealand-Australia	[69]

n.d. not determined
